# The case for targeting enhancer regions in high‐risk TCF3::HLF‐positive B‐cell acute lymphoblastic leukemia

**DOI:** 10.1002/hem3.70442

**Published:** 2026-08-06

**Authors:** Zahra Sheybani, Charles E. de Bock

**Affiliations:** ^1^ Children's Cancer Institute Minderoo Children's Comprehensive Cancer Centre Randwick New South Wales Australia; ^2^ School of Clinical Medicine, Faculty of Medicine UNSW Sydney Sydney New South Wales Australia

Overall survival for children diagnosed with acute lymphoblastic leukemia (ALL) is now above 90%. However, this statistic can be misleading because not all genetic subtypes of ALL patients share in this success. Amongst the worst are children diagnosed with TCF3::HLF‐positive B‐cell ALL (B‐ALL), arising from the translocation t(17;19)(q22;p13). Although rare at less than 1% of cases, these patients have a 5‐year event‐free survival of approximately 25%, respond poorly to induction chemotherapy and essentially fatal at relapse.^1^, ^2^, ^3^ This TCF3::HLF fusion has been shown to rewire the transcriptional program in committed lymphoid progenitors towards a stem‐like, myeloid‐biased state, overwhelmingly by binding to enhancer elements in intronic and intergenic regions rather than at promoters.^2^, ^4^ However, direct links between TCF3::HLF‐bound enhancers and target genes have remained unclear. Furthermore, progress on understanding the early steps of TCF3::HLF‐driven leukemogenesis has in part been due to the inability to generate a genetically engineered mouse model.^5^ Now two new studies^6^, ^7^ provide new mechanistic insight on how the TCF3::HLF fusion drives leukemogenesis and supports cell proliferation.

In the first study by Priebe et al.,^6^ the authors provide a comprehensive three‐dimensional map of the TCF3::HLF regulome. Using TCF3::HLF‐HiChIP in HAL‐01 cells, they demonstrate that 94% of bound sites were within intronic or intergenic regions enriched at chromatin with H3K27ac and H3K4me1 that demarcate enhancers. The authors then ranked the interactions by cumulative contact frequency and found *MEF2C* (myocyte enhancer factor 2C) at the very top of this list. The functional importance of this interaction and *MEF2C* was validated using CRISPR‐mediated disruption of the HLF binding motif within the *MEF2C* intragenic enhancer. This selectively decreased the proliferation of HAL‐01 cells but not in 697 cells harboring the related TCF3::PBX1 fusion. Direct *MEF2C* knockout reproduced the same block in HAL‐01 and a second TCF3::HLF‐positive cell line YCUB‐2 and was equivalent to loss of TCF3::HLF itself. This new data suggests that a single enhancer–promoter interaction in the MEF2C locus sits at the apex of the TCF3::HLF oncogenic hierarchy, sustaining a self‐renewal program as essential to leukemia maintenance as the fusion protein itself (Figure 1A).

**Figure 1:**
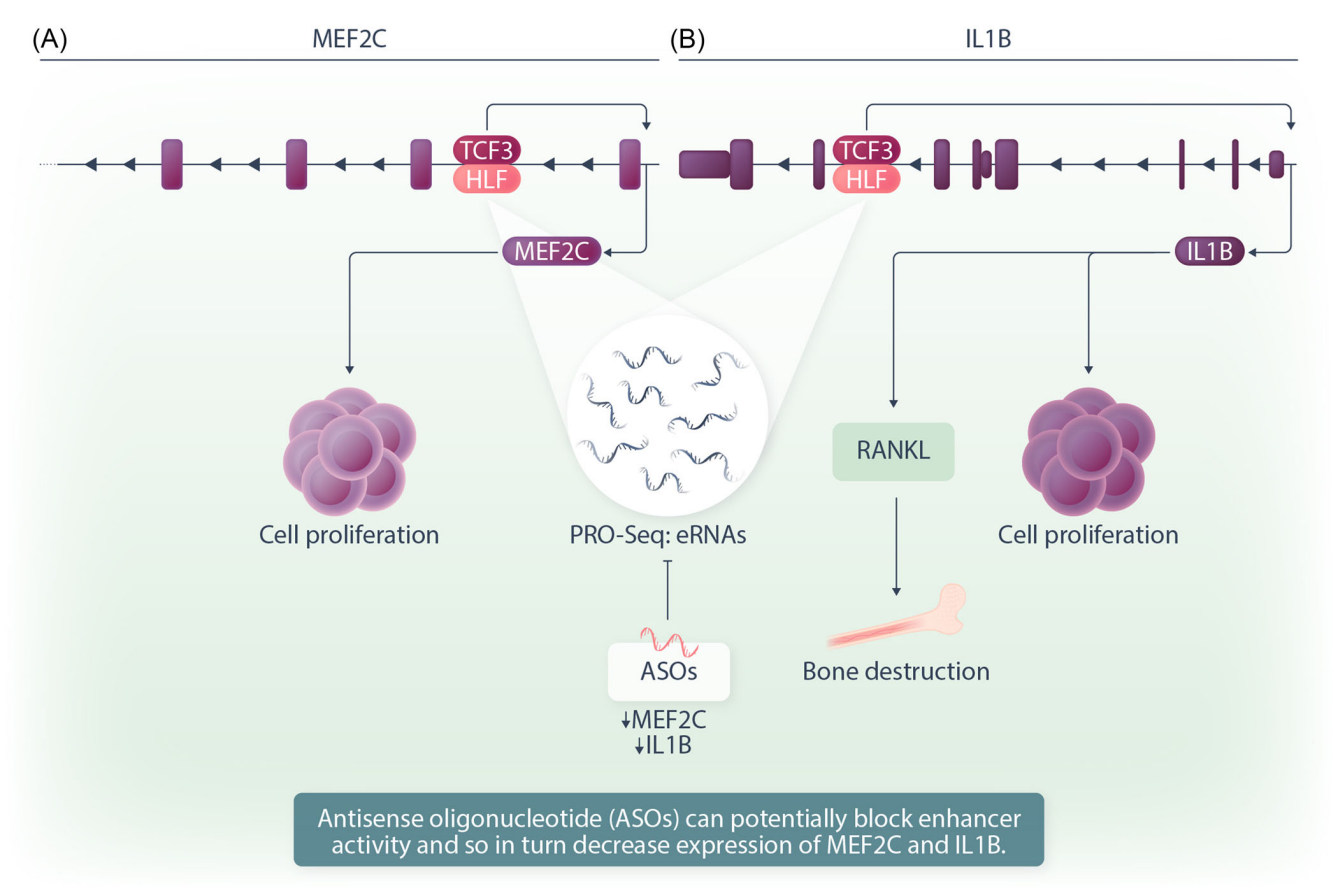
**Schematic of critical enhancer regions bound by the TCF3::HLF fusion at the (A) *MEF2C* locus and (B) *IL1B* locus, illustrating their role in driving proliferation and survival of leukemia cells.** Aberrant activation of *IL1B* is additionally associated with bone destruction. Nascent transcription profiling approaches, such as PRO‐seq, could be used to identify enhancer RNAs (eRNAs) arising from these fusion‐bound regulatory elements. These eRNAs (if present) may represent selective therapeutic targets, where antisense oligonucleotides (ASOs) could be leveraged to suppress *MEF2C* and *IL1B* expression indirectly, thereby mitigating any on target, off tumor toxicity associated with direct inhibition of either MEF2C or IL‐1β.

In the second study by Suzuki et al.,^7^ the authors independently address two other critical knowledge gaps: the lack of a faithful murine model and the unexplained clinical association of TCF3::HLF B‐ALL with hypercalcaemia and osteolytic bone disease. Using induced leukocyte stem cells transduced with TCF3::HLF, the authors generated a mouse model that recapitulates aggressive pro‐B‐like leukemia and also an osteolytic bone phenotype.

Transcriptomic profiling identified interleukin‐1β (IL‐1β) as the most specifically upregulated cytokine in TCF3::HLF versus TCF3::PBX1 B‐ALL. Consistent with the work by Priebe et al., this expression is also driven by an enhancer bound by TCF3::HLF, this time within the intronic region of the IL1B gene locus. This increased IL‐1β expression is implicated in not only increasing cell proliferation but also upregulating receptor activator of nuclear factor κB (NF‐κB) ligand (RANKL) that is responsible for osteoclast activation and the observed bone destruction (Figure 1B). Whilst this second study does not show direct enhancer–promoter looping, indirect evidence comes from CRISPR deletion of this enhancer region that abolished IL‐1β expression and reduced leukemic cell proliferation. Furthermore, IL1B knockout in leukemic blasts transplanted into mice showed prolonged survival, prevented bone destruction, and hematopoietic niche disruption. Taken together, these studies reveal that TCF3::HLF creates at least two distinct, leukemia‐specific regulatory hubs. One centered on MEF2C and one on IL‐1β that are both driven by intragenic enhancer elements uniquely accessible in TCF3::HLF B‐ALL.

How does this new knowledge provide an opportunity to improve the survival of patients diagnosed with TCF3:HLF‐positive B‐ALL? One testable strategy would be to use IL‐1β blocking antibodies that would have the added benefit of addressing the bone disease that now has a mechanistic explanation. Unfortunately, directly targeting MEF2C is more complicated. It is an essential transcription factor for cardiac morphogenesis and cortical neuronal development^8^, ^9^, ^10^ and therefore global MEF2C inhibition would almost certainly be prohibitively toxic.

One exciting development is the potential to target the short non‐coding enhancer RNAs (eRNAs) that are transcribed by active enhancers.^11^, ^12^ These eRNAs would be unique and therefore minimize on‐target off‐tumor toxicity related to direct targeting of MEF2C. Proof‐of‐concept for this strategy comes from an unrelated study also published this year on sickle cell disease. Here, Wang et al.^13^ show that the erythroid‐specific +58 intronic enhancer of *BCL11A*, the same locus targeted by the first approved CRISPR gene therapy for sickle cell disease, expresses unique eRNA transcripts that maintain a higher order chromatin rosette protecting *BCL11A* from Polycomb‐mediated silencing. Depletion of this eRNA using modified antisense oligonucleotides (ASOs) triggers H3K27me3 spreading and silencing of *BCL11A* expression without genome editing, achieving meaningful fetal hemoglobin reactivation in primary erythroblasts from sickle cell patients.

Translating this to the TCF3::HLF context would require the *MEF2C* and *IL1B* enhancer eRNAs to be precisely characterized using techniques such as PRO‐cap and PRO‐seq thereby enabling specific ASOs to be designed (Figure 1). The critical experiment would then be to determine whether these ASO targeting the unique eRNAs phenocopy the anti‐proliferative effect of CRISPR‐mediated enhancer disruption by decreasing *MEF2C* or *IL1B* gene expression. If so, this could then help inform novel combinations that combine eRNA‐directed silencing with small molecule inhibitors such as venetoclax that blocks BCL2 and itself also a direct TCF3::HLF transcriptional target. Perhaps then, this high‐risk subtype might also be associated with overall survival above 90% seen more broadly across B‐ALL.

## AUTHOR CONTRIBUTIONS

**Zahra Sheybani:** Conceptualization; writing original draft; writing review editing. **Charles E. de Bock:** Conceptualization; writing review editing; writing original draft; supervision.

## CONFLICT OF INTEREST STATEMENT

The authors declare no conflicts of interest.

## FUNDING

No funding was received for this publication.

## Data Availability

Data sharing not applicable to this article as no datasets were generated or analyzed during the current study.

## References

[1] Fischer U , Forster M , Rinaldi A , et al. Genomics and drug profiling of fatal TCF3‐HLF‐positive acute lymphoblastic leukemia identifies recurrent mutation patterns and therapeutic options. Nat Genet. 2015;47:1020‐1029. 26214592 10.1038/ng.3362 PMC4603357

[2] Lejman M , Ozygała A , Wróblewska K , Zegardło W , Widz P , Żyła M . A novel approach to understanding the role of TCF3 mutations in childhood B‐cell precursor acute lymphoblastic leukemia. Transl Oncol. 2025;61:102505. 40811980 10.1016/j.tranon.2025.102505 PMC12363590

[3] Arfeuille C , Cavé H . Relevant subtypes in childhood ALL. HemaSphere. 2019;3:174‐177. 35309767 10.1097/HS9.0000000000000245 PMC8925679

[4] Huang Y , Mouttet B , Warnatz HJ , et al. The leukemogenic TCF3‐HLF complex rewires enhancers driving cellular identity and self‐renewal conferring EP300 vulnerability. Cancer Cell. 2019;36:630‐644.e9. 31735627 10.1016/j.ccell.2019.10.004

[5] Huang Y , Marovca B , Dettwiler S , Bode PK , Bornhauser B , Bourquin JP . Rapid generation of leukemogenic chromosomal translocations in vivo using CRISPR/Cas9. HemaSphere. 2020;4:e456. 33134860 10.1097/HS9.0000000000000456 PMC7544329

[6] Priebe V , Galvan B , Drakul A , et al. TCF3::HLF orchestrates an enhancer–promoter network with activation of MEF2C to promote immature HSC gene expression in leukemia. Sci Adv. 2026;12:eadu3728. 42090509 10.1126/sciadv.adu3728 PMC13148343

[7] Suzuki A , Shigehiro T , Hirakawa M , et al. Self‐reinforcing IL‐1β signaling accelerates the development and recurrence of TCF3::HLF‐positive B‐ALL. Blood. 2026;147:2472‐2488. 41774513 10.1182/blood.2025031521

[8] Assali A , Harrington AJ , Cowan CW . Emerging roles for MEF2 in brain development and mental disorders. Curr Opin Neurobiol. 2019;59:49‐58. 31129473 10.1016/j.conb.2019.04.008 PMC6874740

[9] Materna SC , Sinha T , Barnes RM , Lammerts van Bueren K , Black BL . Cardiovascular development and survival require Mef2c function in the myocardial but not the endothelial lineage. Dev Biol. 2019;445:170‐177. 30521808 10.1016/j.ydbio.2018.12.002 PMC6370303

[10] Dong C , Yang XZ , Zhang CY , et al. Myocyte enhancer factor 2C and its directly‐interacting proteins: a review. Prog Biophys Mol Biol. 2017;126:22‐30. 28163053 10.1016/j.pbiomolbio.2017.02.002

[11] Wang Q , Ten Dijke P , Fan C . Deciphering the regulatory landscape of enhancer RNAs in health and disease. Signal Transduct Target Ther. 2026;11:29. 41605892 10.1038/s41392-025-02436-z PMC12852191

[12] Hou TY , Kraus WL . Spirits in the material world: enhancer RNAs in transcriptional regulation. Trends Biochem Sci. 2021;46:138‐153. 32888773 10.1016/j.tibs.2020.08.007 PMC7855021

[13] Wang K , Wang J , Feng R , et al. Silencing of BCL11A by disrupting enhancer‐dependent epigenetic insulation. Blood. 2026;147:1470‐1484. 41191525 10.1182/blood.2025030211 PMC13077484

